# Synthesis and Spectrophotometric Analysis of 1-Azafluorenone Derivatives

**DOI:** 10.3390/molecules25153358

**Published:** 2020-07-24

**Authors:** Nicholas H. Angello, Robert E. Wiley, Christopher J. Abelt, Jonathan R. Scheerer

**Affiliations:** Department of Chemistry, The College of William & Mary, P.O. Box 8795, Williamsburg, VA 23187, USA; nangello@email.wm.edu (N.H.A.); rewiley@email.wm.edu (R.E.W.)

**Keywords:** azafluorenone, synthesis, fluorescence

## Abstract

A new extension for the ‘one pot’ construction of diverse 1-azafluorene derivatives featuring a Diels–Alder/retro-Diels–Alder cycloaddition is reported. Conditions were also determined for oxidation to the derived azafluorenones. The spectrophotometric analysis of five different azafluorenones were performed. Moderate fluorescence was observed with azafluorenone derivatives that bear an imbedded pyridone motif; whereas those bearing substituted pyridines do not fluoresce.

## 1. Introduction

The spectrophotometric properties of fluorenone have been studied for over a century, and the molecule remains of current interest to a diverse community of physical and organic chemists, photochemists and material scientists [1]. The fundamental photophysical properties for the parent 9-fluorenone (**1**) continue to be refined [2] and the resulting enhanced understanding has enabled various applications such as incorporating fluorenone-based molecules for resistive memory devices [3]. Recent advances in modern organic photochemistry have returned to fluorenone as well defined molecule that can in some cases provide superior results as compared to other photosensitizers [4,5]. Several analogs of fluorenone have been prepared with heteroatom substitutions on or within the core. The inclusion of more basic functionalities permits the tuning of the donor–acceptor properties. In particular, 3-aminofluorenone derivatives (e.g., **2**) have been extensively studied and show both an enhanced quantum yield and strong emissive properties stemming from the intramolecular charge transfer excited state [6]. The emission of these derivatives is strongly quenched by hydrogen-bonding interactions with solvents. The mechanism (in plane/out of plane) and stoichiometry of the interactions with the emissive excited state are currently under investigation [7].

**Figure 1:**
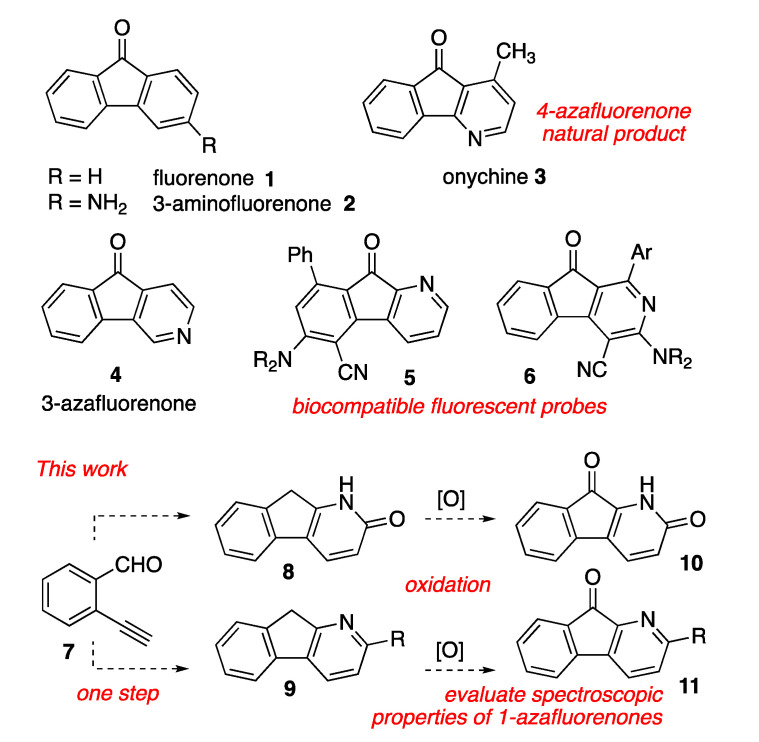
Fluorenone derivatives and the proposed synthesis and evaluation of 1-azafluorenones.

Pyridine analogs of fluorenone have been known for some time as natural products [8]. Renewed isolation efforts have revealed nearly a dozen substituted 4-azafluorenone derivatives related to onychine (**3**), the simplest member of the family [9,10]. Due to potent biological activities, a number of synthetic azafluorenone derivatives have been prepared and show promising therapeutic potential against several diseases and cancer [11,12]. The analysis of the photophysical properties of azafluorenone derivatives have been more limited; 3-azafluorenone **4** has been spectroscopically characterized [13,14] and 1,8-diazafluorenone has been advanced in the field of forensic analysis for the fluorescent illumination of finger print residue [15]. Since the pyridine motif can impart favorable aqueous solubility properties, azafluorenones containing this structural feature have recently emerged as a new class of biocompatible imaging reagents. The 1-aza- and 2-azafluorenone derivatives **5** and **6** are illustrated as representative examples (Figure 1) [16,17].

We recently developed a new synthetic strategy for the construction of 1-azafluorene derivatives **8** and **9** in one reaction vessel from 2-alkynylbenzaldehyde derivatives (e.g., **7**) [18,19]. This manuscript both advances the related methods for the rapid construction of diverse 1-azafluorene structures and determines the photophysical properties of a series of derived azafluoreneones represented by structures **10** and **11**. As such, the products of both the synthetic effort and spectroscopic characterization are described. We were particularly interested in comparing the properties of 1-azafluorenone derivatives bearing either the pyridine structure motif in **11** and the pyridone functionality present in **10**. The pyridone **10** bears an in-plane N–H bond in close proximity to the carbonyl and we wondered what effect such an orientation would have on the optical properties.

## 2. Results and Discussion

### 2.1. Synthesis

In our previous research, we discovered a domino reaction pathway starting from either the diketopiperazine or oxazinone precursors **12** or **13** that enabled a rapid assembly of azafluorene derivatives [18,19]. Although the reaction conditions can vary somewhat for different precursors, both substrates undergo a cascade reaction sequence comprised of an aldol condensation, alkene isomerization, Diels–Alder cycloaddition, and cycloreversion steps (Reactions (1) and (2)). The foundational discovery highlighted in Reaction (1) affords pyridone-containing structures **8** after a base-promoted extrusion of the lactimide bridge. Starting with the lactim-lactone precursor **13**, the derived intermediate [2.2.2]-bicycloadduct is not isolable under the reaction conditions (PhMe, 110 °C) and undergoes the cycloreversion and extrusion of carbon dioxide to reveal the 2-methoxy-1-azafluorene product **9a** (Reaction (2)). The reactivity and scope for these reaction sequences have been disclosed in initial communications. 


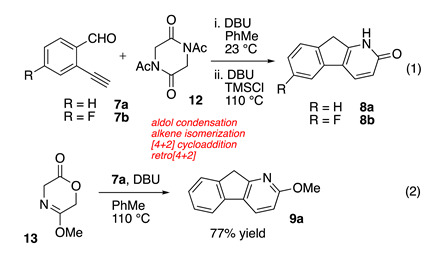


We now wish to report that a modified and analogous reaction sequence with phenyl substituted dihydrooxazinone **14** was achieved using very mild reaction conditions (Reaction (3)). We determined that the entire sequence to give azafluorene product **9b**—the aldol, isomerization, [4+2], and retro-[4+2]—could be accomplished with a non-hygroscopic weak fluoride base (TBAT, tetrabutylammonium difluorotriphenylsilicate) at ambient temperatures and in reliable yields (50–70%). To the best of our knowledge, this is the lowest temperature observed for any cycloaddition/cycloreversion sequence with an oxazinone derivative. In practice, due to the propensity for degradation or polymerization on storage, dihydrooxazinone **14** used in this sequence was prepared in situ via Staudinger reductive cyclization from the derived azide precursor (see Supplementary Materials). Azafluorene prepared in this manner gave more consistent results, on average a 70% yield over the complete operation. Overall, the three related domino reaction sequences highlighted in Reactions (1)–(3) enable the rapid preparation of diverse azafluorene derivatives. 


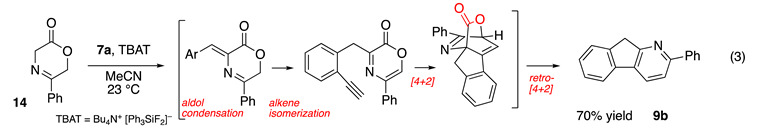


**Scheme 1:**
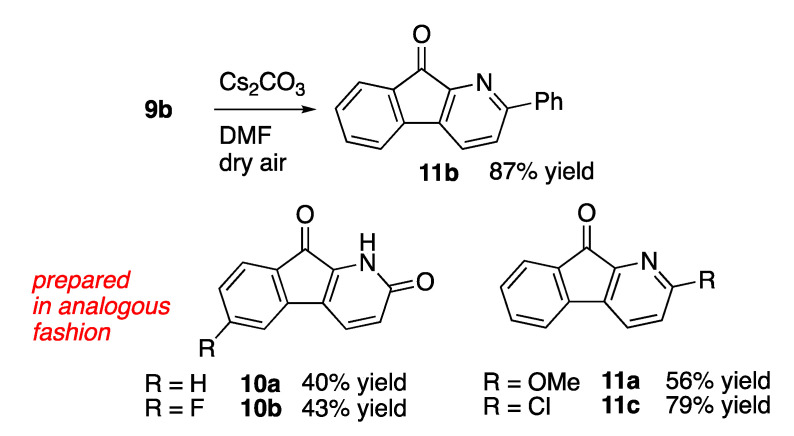
Preparation of the diverse 1-azafluorenones for spectroscopic characterization.

We selected a series of azafluorenes to advance through oxidation to the derived fluorenone products for spectroscopic analysis. In our hands, we found that the fluorenone product **11b** could be most conveniently accessed by treating a DMF solution of the tricyclic fluorene **9b** with Cs_2_CO_3_ and air (Scheme 1). This oxidation proved general and another four products were prepared in an analogous fashion. Thus, five 1-azafluorenone substrates (**10a**, **10b**, **11a**–**11c**) were advanced for spectrophotometric analysis. These 1-azafluoreneone derivatives were selected in order to possess a representative mix of both pyridine and pyridone structural motifs. Additionally, varied substitutions at the C2 position, including carbon (Ph, **11b**), oxygen (OMe, **11a**; O, **10a** and **10b**) and halogen (Cl, **11c**), were included for substrate diversity.

### 2.2. Optical Properties

**Figure 2:**
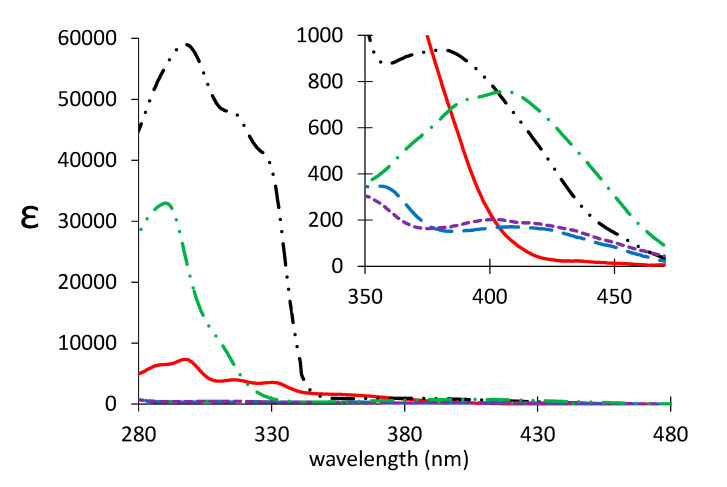
Absorption and long wavelength expansion (inset) of pyridones **10a**,**b** in ethanol and azafluorenones **11a**–**c** in EtOAc. **11c** (Cl), ——; **11b** (Ph), — ^. .^ —; **11a** (MeO), — ^.^ —; pyridone **10a** (H), — —; **10b** (F), – – –.

The electronic absorption spectra of **10a**, **10b**, and **11a**–**11c** are shown in Figure 2. All have the expected strong, short-wavelength UV bands below 300 nm. Methoxyazafluorenone **11a** and the related phenyl derivative **11b** have moderate bands in the medium UV (300–330 nm) and very weak bands around 400 nm, giving the solutions of these compounds a yellow color. The substituted azafluorenones, especially the phenyl derivative, have larger molar absorptivities than the pyridones. The pyridone derivatives **10a** and **10b** show three bands spaced between 300 and 480 nm of similar strength.

**Figure 3:**
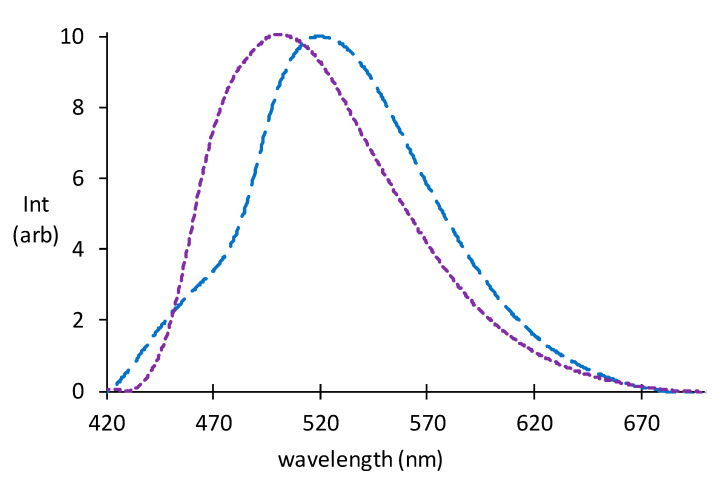
Normalized fluorescence of pyridinones **10a** and **10b** in ethanol (**10a** (H), — —; **10b** (F), – – –).

The pyridone derivatives **10a** and **10b** showed moderate fluorescence in ethanol, while the fluorenones **11a**–**11c** were essentially non-fluorescent (Figure 3). Quantum yields are 0.04 ± 0.01 for **10a**; 0.07 ± 0.01 for **10b.**

### 2.3. Theoretical Calculations

**Figure 4:**
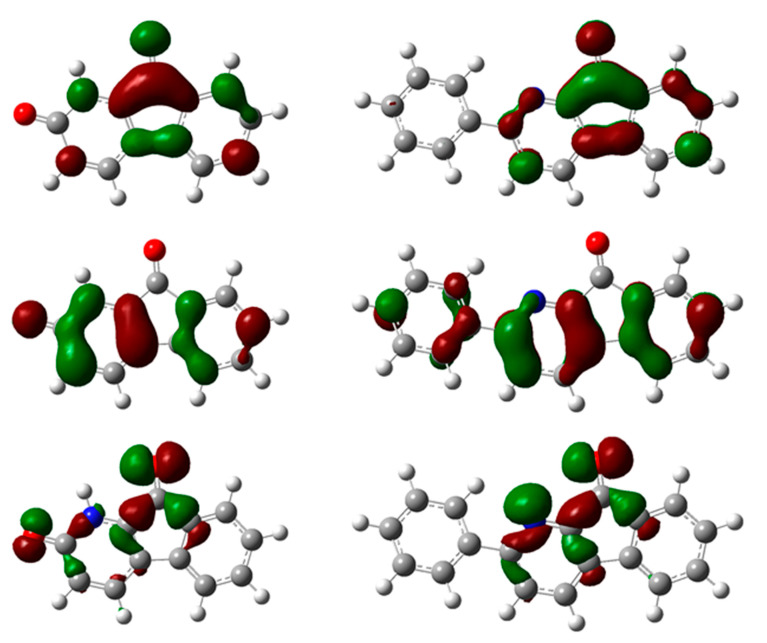
Frontier molecular orbitals (top, LUMO; middle, HOMO; bottom, HOMO-1) for pyridone **10a** (**left**) and phenylazafluorenone **11b** (**right**).

The electronic structures of the ground (DFT, B3YLP, 6-311G+(2d,p)) and the first excited (TD-DFT, B3LYP, 6-31G+(2d,p)) [20] states for **10a**, **10b**, and **11a**–**11c** were calculated with Gaussian 16 [21]. The molecular orbitals for the unsubstituted pyridone **10a** and the phenylazafluorenone **11b** are representative of the two groups. The frontier molecular orbitals, calculated for the ground state structures, are shown in Figure 4. For both molecule sets, the HOMO and LUMO orbitals are π-systems, whereas the HOMO-1 orbital is an *n*-type orbital. Electron density in this orbital includes contributions from the carbonyl oxygen and the pyridine nitrogen. In **11c** and **11a,** the chlorine and methoxy also contribute. For the pyridone set, the HOMO-3 is another *n*-orbital that is centered on the pyridone carbonyl. The calculations predict that strong, short-wavelength (<280 nm) absorption bands were π→π*, whereas the longer wavelength bands have a partial to mostly *n*→π* character (Table 1).

### 2.4. Discussion of Fluorescence

The azafluorenones **11a**–**11c** show no appreciable fluorescence, whereas the pyridones **10a** and **10b** fluoresce abstemiously. Biczόk and coworkers found that rapid intersystem crossing from the lowest singlet excited state (S_1_) to lower energy triplet levels reduced the fluorescence quantum yields of 3-azafluorenone derivatives to the order of 10^−2^ [13,14]. For 1-azafluorenones, **11a**–**11c,** the lowest energy singlet state (S_1_) has *n*→π* character, while the lowest energy triplet state (T_1_) has only π→π* character. These triplet states lie at 11.9, 14.8 and 14.9 kcal/mol lower in energy than the initial S_1_ states.

As a result, the typical spin-orbit coupling mechanism may be responsible for rapid intersystem crossing and explain the relative lack of fluorescence.

**Figure 5:**
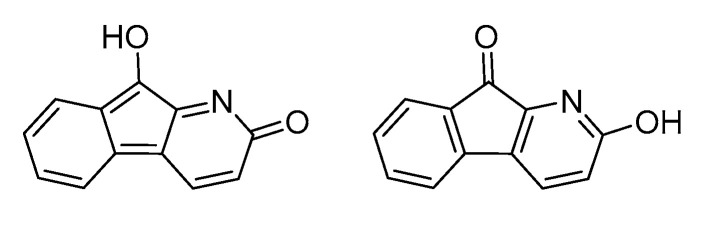
Tautomers of **10a**.

In contrast to the azafluorenones, the pyridones **10a** and **10b** show moderate fluorescence. Here, the π,π* triplet states lie at 37.1 and 36.6 kcal/mol lower in energy than the *n*,π* singlet states. The large energy gap may impede the intersystem crossing. The nature of the fluorescent state for the pyridones is not definite. The structures of the pyridones allow for the possibility of the excited-state intramolecular proton transfer (ESIPT). Proton transfer to the amide carbonyl would give an iminol structure that is similar to **11a** (Figure 5, right). Since **11a** does not show fluorescence, we conclude that this enol structure is not likely to be the source of the pyridone emission. Proton transfer to the fluorenone carbonyl gives the enol structure (Figure 5, left). This structure has an extended, cross-conjugated π-system. For the emission, the calculations predict bands at 504 nm for **10a** and 452 nm for **10b**. However, both of these transitions have some *n*,π* character and zero oscillator strength. For the enol structures, the longest wavelength emission bands are calculated to be at 483 and 489 nm, respectively. These bands are pure π,π* and have non-zero oscillator strengths (both 0.0004). The possibility of ESIPT in the pyridone derivatives remains under investigation.

## 3. Materials and Methods

All reactions were carried out under an atmosphere of nitrogen in flame-dried or oven-dried glassware with magnetic stirring unless otherwise indicated. Acetonitrile, THF, toluene, and Et_2_O were degassed with argon and purified by passage through a column of molecular sieves and a bed of activated alumina [22]. Dichloromethane was distilled from CaH_2_ prior to use. All reagents were used as received unless otherwise noted. Flash column chromatography was performed using SiliCycle siliaflash P60 silica gel (230–400 mesh) [23]. Analytical thin layer chromatography was performed on SiliCycle 60Å glass plates. Visualization was accomplished with UV light, anisaldehyde, ceric ammonium molybdate, potassium permanganate, or ninhydrin, followed by heating. Infrared spectra were recorded using a Digilab FTS 7000 FTIR spectrophotometer. ^1^H-NMR spectra were recorded on a Varian Mercury 400 (400 MHz) spectrometer and are reported in ppm using solvent as an internal standard (CDCl_3_ at 7.26 ppm) or tetramethylsilane (0.00 ppm). Proton-decoupled ^13^C-NMR spectra were recorded on a Mercury 400 (100 MHz) spectrometer and are reported in ppm using solvent as an internal standard (CDCl_3_ at 77.00 ppm). All compounds were judged to be homogeneous (>95% purity) by ^1^H and ^13^C NMR spectroscopy, unless otherwise noted. Mass spectra data analysis was obtained through positive electrospray ionization (w/ NaCl) on a Bruker 12 Tesla APEX–Qe FTICR-MS with an Apollo II ion source. Absorption and fluorescence data were obtained using a fiber optic system with an Ocean Optics Maya CCD detector, a miniature deuterium/tungsten lamp (uv/vis) and a 365 nm LED light source (fluorescence). Relative quantum yields were determined using anthracene as the reference (Φ = 0.30). The emission intensity data was treated as follows: (1) the electronic noise was subtracted, (2) the wavelength values were converted to wavenumbers, (3) the corresponding net intensity values were multiplied by λ^2^/(λ_max_)^2^ to account for the effect of the abscissa-scale transformation and (4) the resulting intensity values were divided by the spectral response of the Hamamatsu S10420 CCD. Electronic structure calculations were conducted using Gaussian 16. Ground state geometries were optimized using the DFT B3YLP method employing the 6-311G+(2d,p) basis set. Excited states were optimized using the TD-DFT B3LYP method employing the 6-31G+(2d,p) basis set.

## 4. Conclusions

We successfully prepared several 1-azafluorenones and related pyridones using an aldol/isomerization/Diels–Alder cycloaddition/cycloreversion cascade reaction sequence followed by fluorene oxidation. The photophysical properties of these compounds were characterized and modeled through computations. The pyridones fluoresce moderately, but the azafluorenones bearing the pyridine motif do not.

## Figures and Tables

**Table 1:** The calculated absorption bands (λ_max_ (nm), oscillator strength, % *n*→π* character) for **10** and **11**.

Compound							
**11c** (Cl)	λ_max_	277	286	301	306		
	*f* × 10^4^	4489	4	4	123		
	% *n*	0	100	100	58		
**11b** (Ph)	λ_max_	288	306	308	316	322	327
	*f* × 10^4^	4995	21	59	423	3219	907
	% *n*	0	0	0	45	0	52
**11a** (MeO)	λ_max_	279	279	303	306	427	
	*f* × 10^4^	4040	3	6	471	1	
	% *n*	0	100	100	0	100	
**10a** (H)	λ_max_	295	302	324	349	403	
	*f* × 10^4^	3065	743	4	0	0	
	% *n*	0	0	0	100	100	
**10b** (F)	λ_max_	291	306	322	347	399	
	*f* × 10^4^	3042	1006	194	0	0	
	% *n*	0	44	41	64	36	

## Supplementary Materials

The following are available online: the experimental procedures, characterization data, ^1^H and the ^13^C-NMR spectra for all new compounds.

## References

[1] Herkstroeter W.G., Hammond G.S., Lamola A.A. (1964). Mechaisms of Photochemical Reactions in Solution. 28. Values of Triplet Excitation energies of Selected Sensitizers. J. Am. Chem. Soc..

[2] Chang C.W., Solling T.I., Diau E.W.G. (2017). Revisiting the photophysics of 9-fluorenone: Ultrafast time-resolved fluorescence and theoretical studies. Chem. Phys. Lett..

[3] Jia M., Li Y., Huang M., Kim K.J., Liu Y., Cao S. (2020). Fluorenone-based molecules for resistive memory devices: Tuning memory behavior by adjusting end groups. Synth. Met..

[4] Xia J.B., Zhu C., Chen C. (2013). Visible Light-Promoted Metal-Free C-H Activation: Diarylketone-Catalyzed Selective Benzylic Mono-and Difluorination. J. Am. Chem. Soc..

[5] Pitts C.R., Bloom M.S., Bume D.D., Zhang Q.A., Lectka T. (2015). Unstrained C-C bond activation and directed fluorination through photocatalytically-generated radical cations. Chem. Sci..

[6] Biczόk L., Berces T., Inoue H. (1999). Effects of molecular structure and hydrogen bonding on the radiationless deactivation of singlet excited fluorenone derivatives. J. Phys. Chem..

[7] Alty I.G., Abelt C.J. (2017). Stereoelectronics of the Hydrogen-Bond-Induced Fluorescence Quenching of 3-Aminofluorenones with Alcohols. J. Phys. Chem. A.

[8] De Almeida M.E.L., Braz-F R., Von Bulow M.V., Gottlieb O.R., Maia J.G.S. (1976). Onychine, an alkaloid from *Onychopetalum amazonicum*. Phytochemistry.

[9] Pumsalid K., Thaisuchat H., Loetchutinat C., Nuntasaen N., Meepowpan P., Pompimon W. (2010). A New Azafluorenone from the Roots of Polyalthia cerasoides and its Biological Activity. Nat. Prod. Commun..

[10] Mueller D., Davis R.A., Duffy S., Avery V.M., Camp D., Quinn R.J. (2009). Antimalarial Activity of Azafluorenone Alkaloids from the Australian Tree Mitrephora diversifolia. J. Nat. Prod..

[11] Shi Y.B., Gao S.H. (2016). Recent advances of synthesis of fluorenone and fluorene containing natural products. Tetrahedron.

[12] Ye F., Tran C., Jullien L., Le Saux T., Haddad M., Michelet V., Ratovelomanana-Vidal V. (2018). Synthesis of Fluorescent Azafluorenones and Derivatives via a Ruthenium-Catalyzed 2+2+2 Cycloaddition. Org. Lett..

[13] Biczόk L., Cser A., Nagy K. (2001). Substituent and solvent effects on the photophysical properties of 3-azafluorenone derivatives. J. Photochem. Photobiol. A Chem..

[14] Biczόk L. (1997). Photophysical properties of 3-azafluorenone. React. Kinet. Catal. Lett..

[15] Grigg R., Mongkolaussavaratana T., Pounds C.A., Sivagnanam S. (1990). 1,8-Diazafluorenone and related-compounds-a new reagent for the detection of alpha-amino-acids and latent fingerprints. Tetrahedron Lett..

[16] Gao M., Su H.F., Lin Y.H., Ling X., Li S.W., Qin A.J., Tang B.Z. (2017). Photoactivatable aggregation-induced emission probes for lipid droplets-specific live cell imaging. Chem. Sci..

[17] Sharma A., Umar S., Kar P., Singh K., Sachdev M., Goel A. (2016). A new type of biocompatible fluorescent probe AFN for fixed and live cell imaging of intracellular lipid droplets. Analyst.

[18] Angello N.H., Wiley R.E., Elmore T.G., Perry R.S., Scheerer J.R. (2018). Domino Reaction Sequence for the Synthesis of 2.2.2 Diazabicycloalkenes and Base-Promoted Cycloreversion to 2-Pyridone Alkaloids. Org. Lett..

[19] Williamson J.B., Smith E.R., Scheerer J.R. (2017). A Merged Aldol Condensation, Alkene Isomerization, Cycloaddition/Cycloreversion Sequence Employing Oxazinone Intermediates for the Synthesis of Substituted Pyridines. Synlett.

[20] Foresman J.B., Frish A.E. (2015). Exploring Chemistry with Electronic Structure Methods.

[21] Frisch M.J., Trucks G.W., Schlegel H.B., Scuseria G.E., Robb M.A., Cheeseman J.R., Scalmani G., Barone V., Petersson G.A., Nakatsuji H. (2016). Gaussian 16.

[22] Pangborn A.B., Giardello M.A., Grubbs R.H., Rosen R.K., Timmers F.J. (1996). Safe and Convenient Procedure for Solvent Purification. Organometallic.

[23] Still W.C., Kahn M., Mitra A. (1978). Rapid chromatographic technique for preparative separations with moderate resolution. J. Org. Chem..

