# Supplementary material for: Synthesis and Spectrophotometric Analysis of 1-Azafluorenone Derivatives
Source: Molecules. 2020 Jul 24;25(15):3358. doi: 10.3390/molecules25153358 (PMC7436005; doi:10.3390/molecules25153358)
Supplement: Supplementary file 1 [file molecules-25-03358-s001.pdf]

Synthesis and spectrophotometric analysis of 1-azafluorenone derivatives

Nicholas H. Angello, Robert E. Wiley, Christopher J. Abelt* and Jonathan R. Scheerer *

Department of Chemistry, The College of William & Mary, P.O. Box 8795, Williamsburg, Virginia 23187, United States

* Correspondence: cjabel@wm.edu; jrscheerer@wm.edu

Supporting Information

1. General Information S1

2. Experimental Procedures and Characterization Data S2–S4

3. ^{1}H and ^{13}C NMR Spectra S5–S11

General Information. All reactions were carried out under an atmosphere of nitrogen in flame-dried or oven-dried glassware with magnetic stirring unless otherwise indicated. Acetonitrile, THF, toluene, and Et_2O were degassed with argon and purified by passage through a column of molecular sieves and a bed of activated alumina.[1] Dichloromethane was distilled from CaH_2 prior to use. All reagents were used as received unless otherwise noted. Flash column chromatography[2] was performed using SiliCycle siliaflash P60 silica gel (230–400 mesh). Analytical thin layer chromatography was performed on SiliCycle 60Å glass plates. Visualization was accomplished with UV light, anisaldehyde, ceric ammonium molybdate, potassium permanganate, or ninhydrin, followed by heating. Film infrared spectra were recorded using a Digilab FTS 7000 FTIR spectrophotometer. ^{1}H NMR spectra were recorded on a Varian Mercury 400 (400 MHz) spectrometer and are reported in ppm using solvent as an internal standard (CDCl3 at 7.26 ppm) or tetramethylsilane (0.00 ppm). Proton-decoupled ^{13}C-NMR spectra were recorded on a Mercury 400 (100 MHz) spectrometer and are reported in ppm using solvent as an internal standard ($CDCl_3$ at 77.00 ppm). All compounds were judged to be homogeneous (>95% purity) by ^{1}H and ^{13}C NMR spectroscopy, unless otherwise noted. Mass spectra data analysis was obtained through positive electrospray ionization (w/ NaCl) on a Bruker 12 Tesla APEX–Qe FTICR-MS with an Apollo II ion source. Absorption and fluorescence data were obtained using a fiber optic system with an Ocean Optics Maya CCD detector, a miniature deuterium/tungsten lamp (uv/vis) and a 365 nm LED light source (fluorescence). Relative quantum yields were determined using anthracene as the reference (Φ = 0.30). The emission intensity data was treated as follows: 1) the electronic noise was subtracted, 2) the wavelength values were converted to wavenumbers, 3) the corresponding net intensity values were multiplied by $\lambda^2/(\lambda_{max})^2$ to account for the effect of the abscissa-scale transformation and 4) the resulting intensity values were divided by the spectral response of the Hamamatsu S10420 CCD. Electronic structure calculations were conducted using Gaussian 16. Ground state geometries were optimized using the DFT B3YLP method employing the 6-311G+(2d,p) basis set. Excited states were optimized using the TD-DFT B3LYP method employing the 6-31G+(2d,p) basis set.

---

1 Pangborn, A. B.; Giardello, M. A.; Grubbs, R. H.; Rosen, R. K.; Timmers, F. J., *Organometal.* **1996,** *15*, 1518-1520.

2 Still, W. C.; Kahn, M.; Mitra, A. *J. Org. Chem.* **1978**, *43*, 2923-2925.

Experimental procedures and characterization data

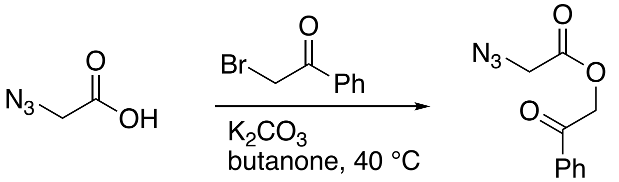

2-oxo-2-phenylethyl 2-azidoacetate. To azidoacetic acid (0.642 g, 6.35 mmol) in butanone (15 mL) was added K_2CO_3 (1.32 g, 9.53 mmol) and 2-bromoacetophenone (1.90 g, 9.53 mmol). The reaction vessel was heated at 40 °C. After stirring for 16 h, the reaction mixture was cooled to rt, diluted with Et_2O, filtered. Water was added to the filtrate and the organic layer was removed. The aqueous layer was extracted with additiona Et_2O (2 x 30 mL). The combined organic layers were washed with brine (25 mL), dried (Na_2SO_4), filtered, and concentrated *in vacuo*. Purification by flash column chromatography on silica gel (gradient elution: 10% to 40% EtOAc in hexanes) afforded 2-oxo-2-phenylethyl 2-azidoacetate (0.940 g, 4.425 mmol, 70% yield) as an amorphous white solid: TLC (20 % EtOAc in hexane), R*f*: 0.25 (CAM); IR 2924, 2100, 1749, 1697, 1204, 972, 731, 689 cm^{-1}; ^{1}H NMR (400 MHz, $CDCl_3$) δ 7.91 (d, J = 7.5 Hz, 2 H), 7.64 (t, J = 7.5 Hz, 1H), 7.51 (apparent triplet, J = 7.6 Hz, 2H), 5.47 (s, 2H), 4.10 (s, 2H); ^{13}C NMR (100 MHz, $CDCl_3$) δ 191.0, 167.9, 134.2, 133.8, 129.0, 127.8, 66.8, 50.1.

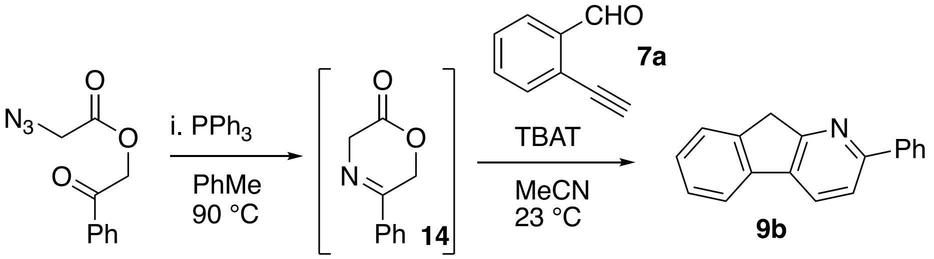

2-phenyl-1-azafluorene, 2-phenyl-9H-indeno[2,1-b]pyridine (9b). A dry flask was charged with 2-oxo-2-phenylethyl 2-azidoacetate (100 mg, 0.46 mmol) and dissolved in PhMe (3 mL). To the solution was added PPh_3 (122 mg, 0.46 mmol). After 5 min at ambient temperatures, the reaction heated to 90 °C. After 9 h, the reaction was cooled to rt and 2-ethynylbenzaldehyde **7a** (66 mg, 0.51 mmol) and tetrabutylammonium difluorotriphenylsilicate (TBAT) (275 mg, 0.51 mmol) were added sequentially. After stirring at rt for 22 h, the reaction was diluted with toluene (10 mL) and ethyl acetate (10 mL) and filtered through a small pad (~1 cm) of silica prior to concentration *in vacuo*. The resulting residue was purified by flash chromatography on silica gel (gradient elution: 0% to 15% EtOAc in hexane) to afford azafluorene **9b** (78 mg, 0.32 mmol, 70% yield) as a colorless solid: mp 161–163 °C; TLC (20% EtOAc in hexane), R*f*: 0.6 (UV, CAM); IR (film) 1393, 837, 754 cm^{-1}; ^{1}H NMR (400 MHz, $CDCl_3$) δ 8.05 (apparent doublet, J = 7.7 Hz, 3H), 7.79 (d, J = 7.3 Hz, 1H), 7.72 (d, J = 8.1 Hz, 1H), 7.61 (d, J = 7.2 Hz, 1H), 7.53-7.46 (m, 2H), 7.45-7.35 (m, 3H), 4.08 (s, 2H); ^{13}C NMR (100 MHz, $CDCl_3$) δ 165.8, 155.8, 141.6, 139.8, 139.2, 133.6, 128.8, 128.7, 127.7, 127.5, 127.1, 127.0, 125.3, 120.6, 118.9, 38.9; Exact mass for $C_{18}H_{14}N[M+H^+]$, 244.1126, found 244.1118.

General procedure for oxidation of azafluorenes to azafluorenones (10–11). To a solution of azafluorenes (1 equiv, 0.1–1.6 mmol) in DMF (0.2M) was added Cs_2CO_3 (3 equiv) in one lot. The vessel was capped with a drying tube (drierite) and allowed to stir open to air. After stirring for 16

h, the reaction was diluted with H_2O (15 mL) and extracted with CH_2Cl_2 (3 x 10 mL). The combined organic layers were washed with brine (10 mL), dried (Na_2SO_4), filtered, and concentrated *in vacuo*.

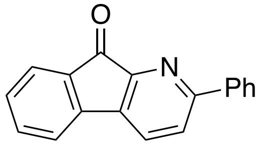

11b

2-phenyl-9*H*-indeno[2,1-*b*]pyridin-9-one (11b). Prepared according to general oxidation procedure with **9b** (28 mg, 0.11 mmol) and afforded compound **11b** (25 mg, 0.10 mmol, 87% yield) as a yellow solid which was >95% pure as judged by ^{1}H NMR. mp 177-179 °C; TLC (20% EtOAc/hexanes) R*f* = 0.30 (Visible light, UV, CAM); IR 1727, 1603, 1450, 1302 cm^{-1}; ^{1}H NMR (400MHz, $CDCl_3$): δ 8.05 (d, *J* = 7.2 Hz, 2H), 7.86 (d, *J* = 8 Hz 1H), 7.74 (d, *J* = 8 Hz, 1H), 7.71 (d, *J* = 7.2 Hz, 1H), 7.49 (m, 5H), 7.33 (t, *J* = 6.8 Hz, 1H); ^{13}C NMR (100 MHz, $CDCl_3$): δ 192.8, 158.4, 153.3, 141.7, 138.4, 138.1, 135.5, 132.6, 129.8, 129.5, 128.8, 127.0, 124.8, 123.8, 121.0; Exact mass for $C_{18}H_{11}NONa$ $[M+Na]_+$, 280.0733, found 280.0729.

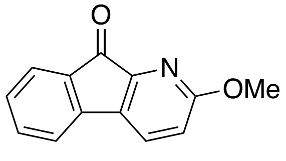

11a

2-methoxy-9*H*-indeno[2,1-*b*]pyridin-9-one (11a). Prepared according to general oxidation procedure with **9a** [3] (21.8 mg, 0.11 mmol) and afforded compound **11a** (12.2 mg, 0.06 mmol, 56% yield) as a yellow solid which was >95% pure as judged by ^{1}H NMR. mp 136-138 °C; TLC (50% $CHCl_3$/hexane) R*f* = 0.30 (Visible light, UV, CAM); IR: 1726, 1306, 1258, 843 cm^{-1}; ^{1}H NMR (400MHz, $CDCl_3$): δ 7.72 (d, *J* = 8.0 Hz, 1H), 7.62 (d, *J* = 7.1 Hz, 1H), 7.45 (t, *J* = 7.5 Hz, 1H), 7.34 (d, *J* = 7.1 Hz, 1H), 7.24 (t, J = 7.5 Hz, 1H), 6.81 (d, *J* = 8.0 Hz, 1H), 4.04 (s, 3H); ^{13}C NMR (100 MHz, $CDCl_3$): δ 192.9, 165.9, 150.6, 142.0, 135.2, 134.8, 131.9, 130.5, 128.6, 124.6, 119.7, 115.5, 54.3; Exact mass for $C_{13}H_9NO_2Na$ $[M+Na]^+$, 234.0525, found 234.0524.

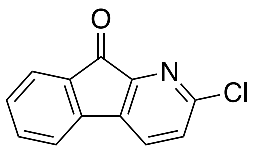

11c

2-chloro-9H-indeno[2,1-b]pyridin-9-one (11c). Prepared according to general oxidation procedure with **9c** [4] (54.5 mg, 0.23 mmol) and afforded compound **11c** (39 mg, 0.18 mmol, 79% yield) as an off-white solid which was >95% pure as judged by ^{1}H NMR. mp 188-190 °C; TLC

[3] Williamson, J.B.; Smith, E.R.; Scheerer, J.R. A Merged Aldol Condensation, Alkene Isomerization, Cycloaddition/Cycloreversion Sequence Employing Oxazinone Intermediates for the Synthesis of Substituted Pyridines. *Synlett* **2017**, *28*, 1170-1172, doi:10.1055/s-0036-1588729.

[4] Angello, N.H.; Wiley, R.E.; Elmore, T.G.; Perry, R.S.; Scheerer, J.R. Domino Reaction Sequence for the Synthesis of 2.2.2 Diazabicycloalkenes and Base-Promoted Cycloreversion to 2-Pyridone Alkaloids. *Organic Letters* **2018**, *20*, 5203-5207, doi:10.1021/acs.orglett.8b02145.

(5% MeOH/$CHCl_3$) R*f* = 0.64 (UV, CAM); IR: 1709, 1587, 1556, 1431, 1412, 1177, 1150, 914, 738 cm^{-1}; ^1H NMR (400MHz, $CDCl_3$): δ 8.53 (d, *J* = 4.9 Hz, 1H), 7.76 (d, *J* = 7.4 Hz, 1H), 7.63 (m, 2H), 7.51 (t, *J* = 7.4 Hz, 1H), 7.46 (d, *J* = 4.9 Hz, 1H); ^{13}C NMR (100 MHz, $CDCl_3$) δ 189.5, 155.5, 155.4, 147.9, 140.1, 135.1, 133.6, 132.1, 124.9, 124.8, 121.9, 114.6; Exact mass for $C_{12}H_6ClNONa$ $[M+Na]^+$, 238.0030, found 238.0031.

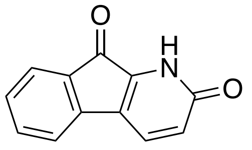

10a

1H-indeno[2,1-b]pyridine-2,9-dione (10a). Prepared according to general oxidation procedure with **8a** [4] (0.30 g, 1.64 mmol) and afforded compound **10a** (130 mg, 0.64 mmol, 40% yield) as a brown solid which was >95% pure as judged by ^1H NMR. mp 274-275 °C; TLC (5% MeOH / $CHCl_3$) R*f* = 0.26 (UV, CAM); IR 1719, 1620, 1586, 1548, 1175, 1023, 994, 749, 689 cm^{-1}; 1H NMR (400MHz, DMSO-*d*6): δ 12.3 (br s, 1H), 7.96 (d, *J* = 6.3 Hz, 1H), 7.86 (d, *J* = 7.2 Hz, 1H), 7.63 (t, *J* = 7.2 Hz, 1H), 7.55 (m, 2H), 6.90 (d, *J* = 6.3 Hz, 1H); ^{13}C NMR (100 MHz, DMSO-*d*6): δ 190.6, 162.0, 157.5, 146.8, 139.5, 134.3, 133.9, 132.3, 123.2, 123.1, 116.3, 99.9; Exact mass for $C_{12}H_7NO_2Na$ $[M+Na]^+$ 220.0369, found 220.0369.

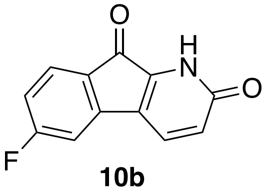

10b

6-fluoro-1H-indeno[2,1-b]pyridine-2,9-dione (10b). Prepared according to general oxidation procedure with **8b** [4] (26 mg, 0.13 mmol) and afforded compound **10a** (12.3 mg, 0.06 mmol, 43% yield) as a dark yellow solid which was >95% pure as judged by ^1H NMR. mp 324-326 °C; TLC (10% MeOH/$CHCl_3$) R*f* = 0.38 (UV, $KMnO_4$); IR 3090, 1707, 1641, 1587, 1471, 1190, 795 cm^{-1}; ^1H NMR (400MHz, DMSO-*d*6): δ 12.34 (s, 1H), 7.99 (d, *J* = 6.3 Hz, 1H), 7.84 (dd, J_1 = 8.4 Hz, J_2 = 2.2 Hz, 1H), 7.61 (dd, J_1 = 8.4 Hz, J_2 = 5.4 Hz, 1H), 7.32 (m, 1H), 6.93 (d, *J* = 6.3 Hz, 1H); ^{13}C NMR (100 MHz, DMSO-*d*6): δ 188.5, 165.7, 159.8, 156.8, 146.5, 142.2, 129.6, 124.9, 117.8, 116.5, 110.9, 99.6; Exact mass for $C_{12}H_6FNO_2Na$ $[M+Na]^+$ 238.0274, found 238.0273.

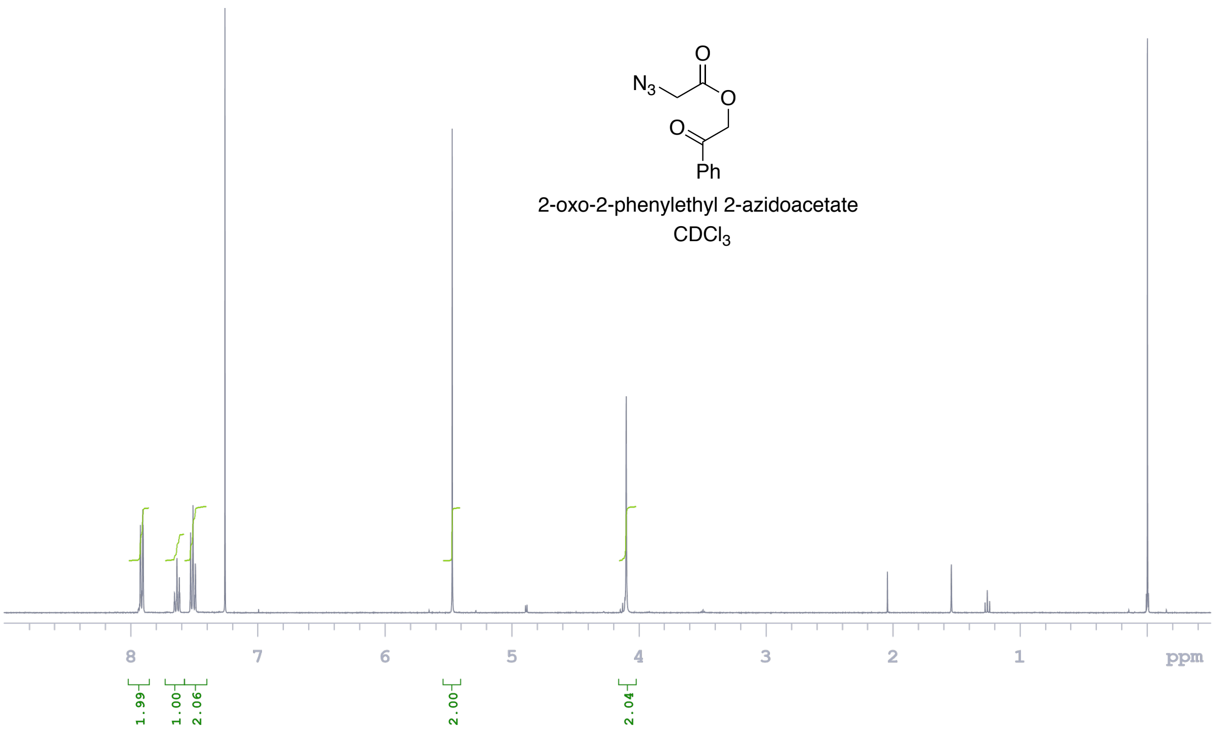

O
N_3
O
O
Ph
2-oxo-2-phenylethyl 2-azidoacetate
$CDCl_3$
1.99
1.00
2.06
2.00
2.04
8
7
6
5
4
3
2
1
ppm

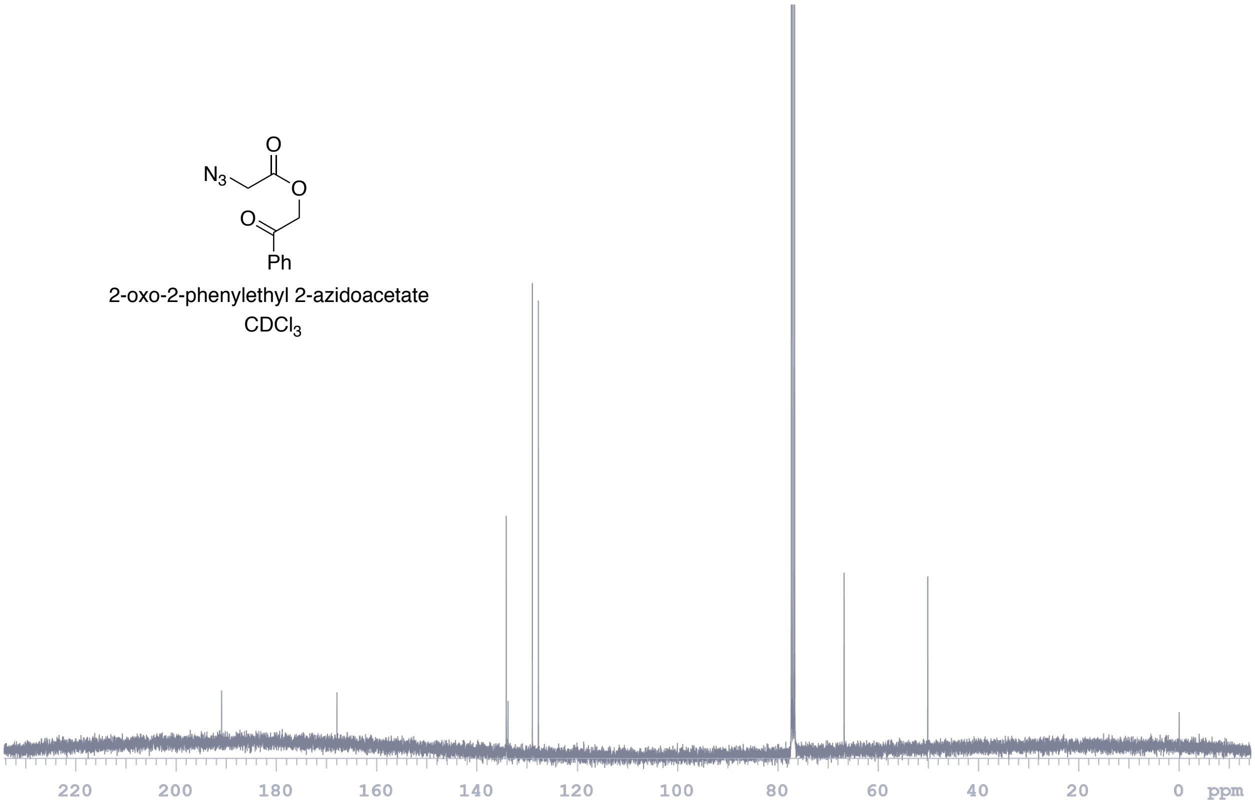

O
N_3
O
O
Ph
2-oxo-2-phenylethyl 2-azidoacetate
$CDCl_3$
220
200
180
160
140
120
100
80
60
40
20
0
ppm

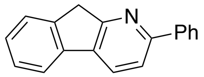

9b

$CDCl_3$

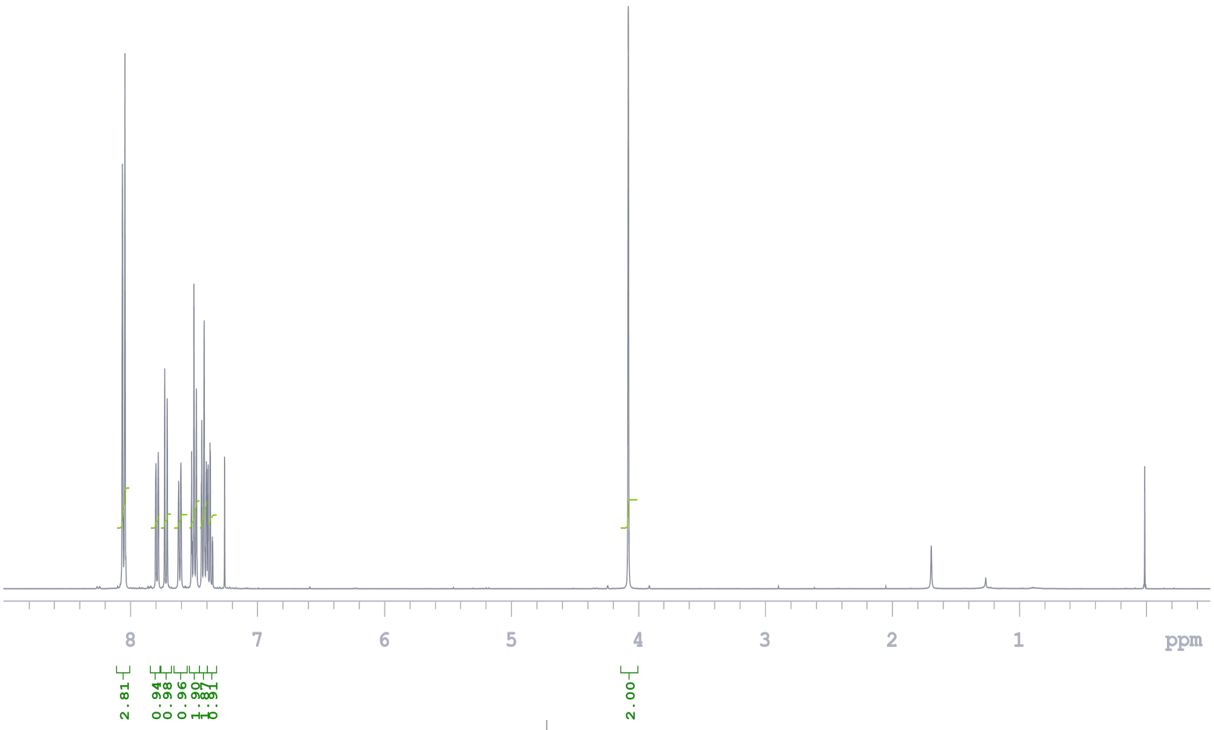

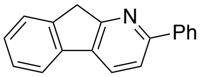

9b

$CDCl_3$

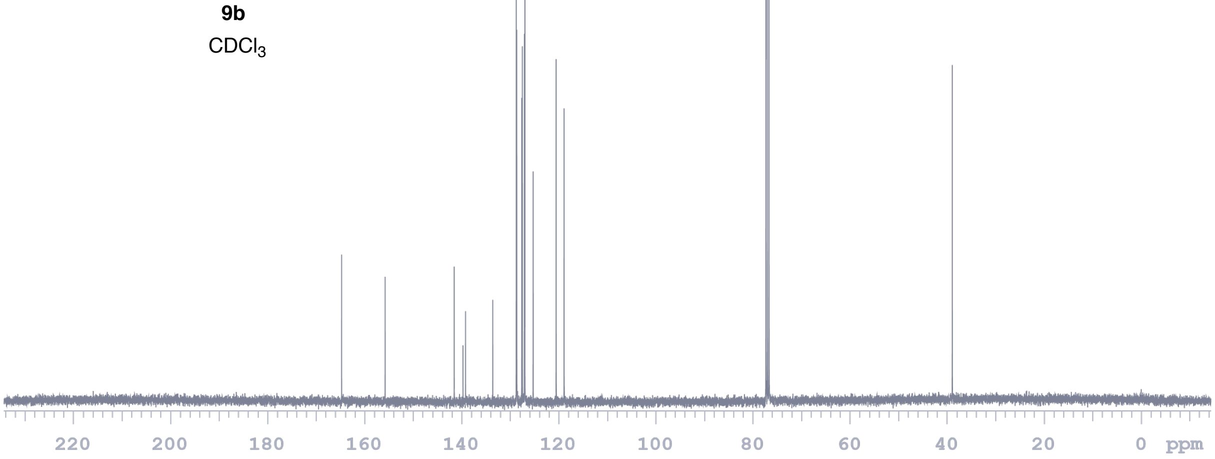

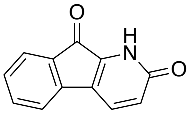

10a

DMSO-d_6

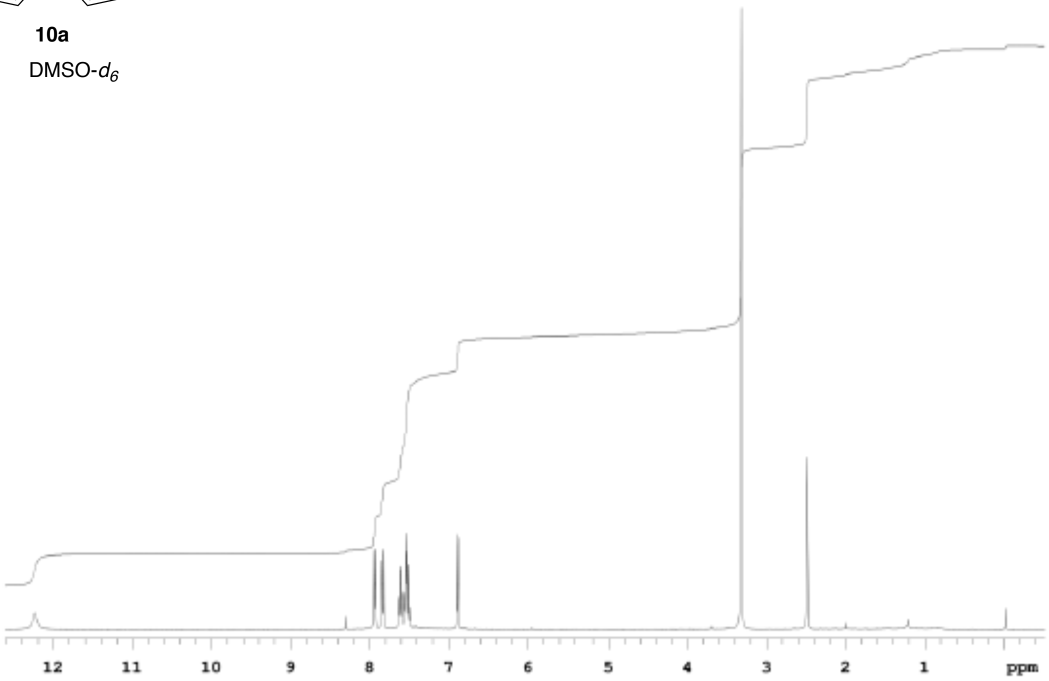

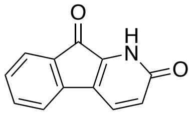

10a

DMSO-d_6

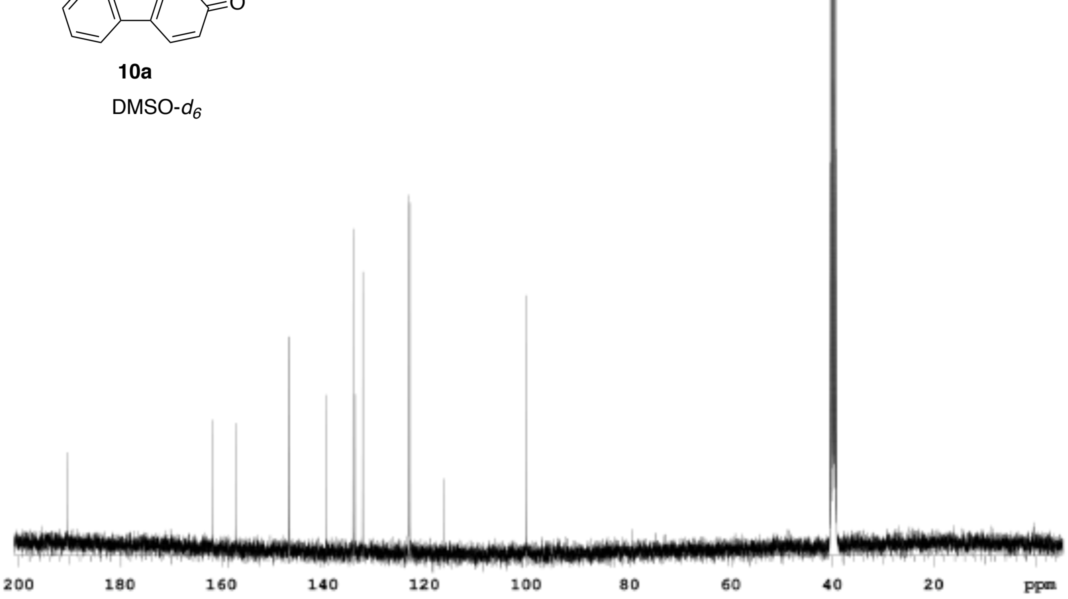

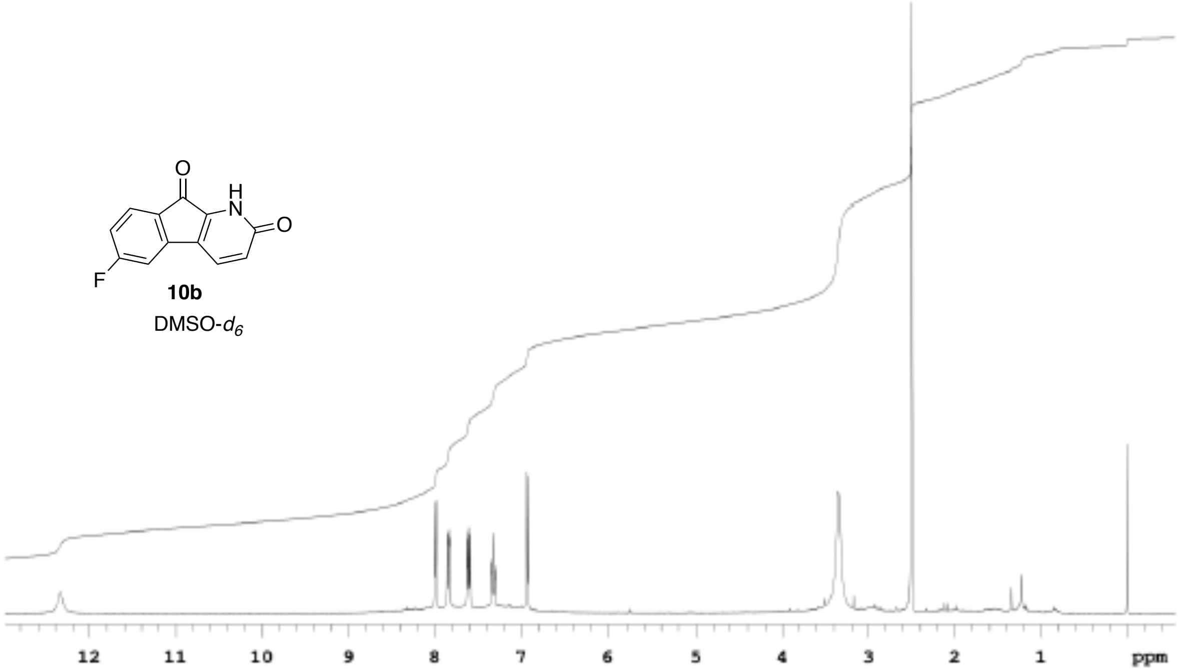
O
H
N
O
F
10b
DMSO-d6
12 11 10 9 8 7 6 5 4 3 2 1 ppm

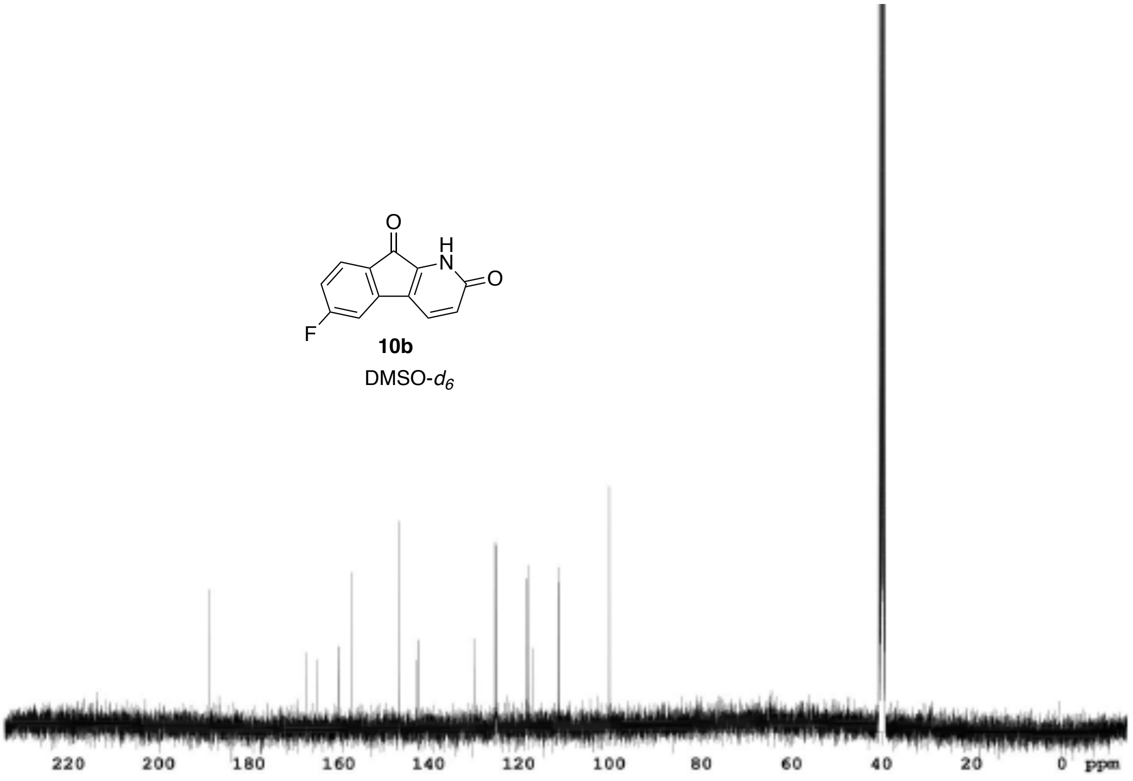
O
H
N
O
F
10b
DMSO-d6
220 200 180 160 140 120 100 80 60 40 20 0 ppm

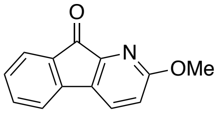

11a
$CDCl_3$

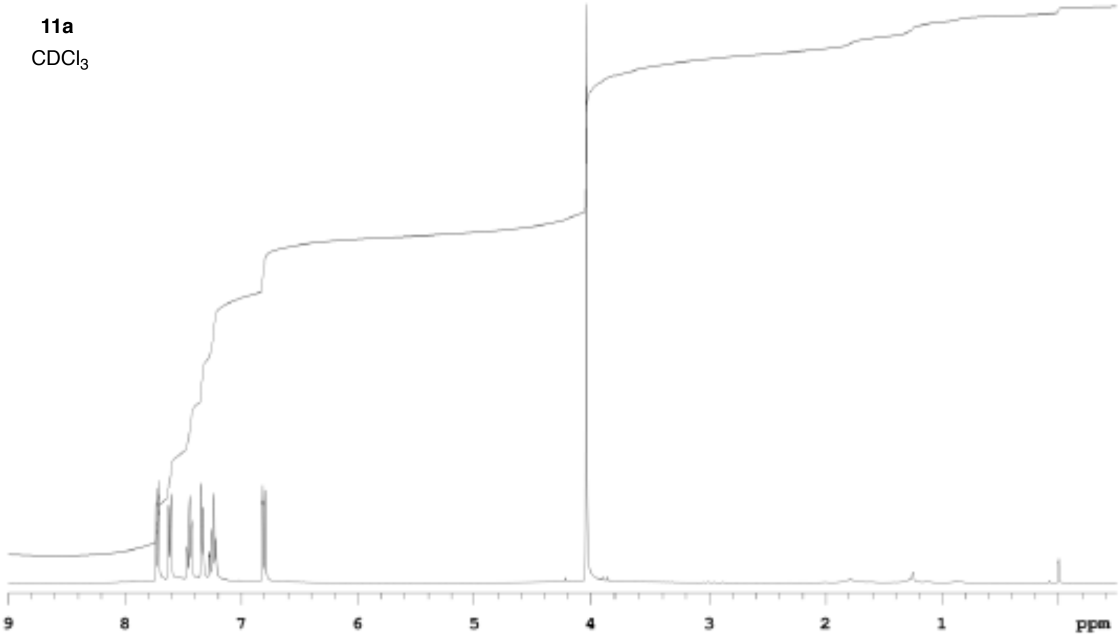

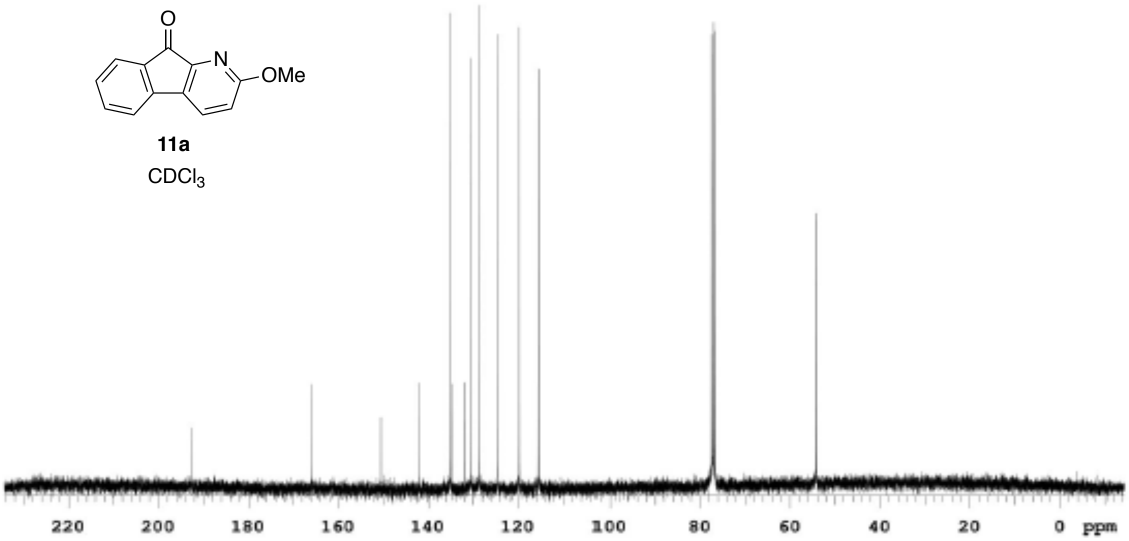

11a
$CDCl_3$

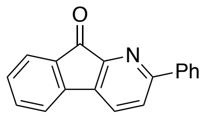

11b
$CDCl_3$

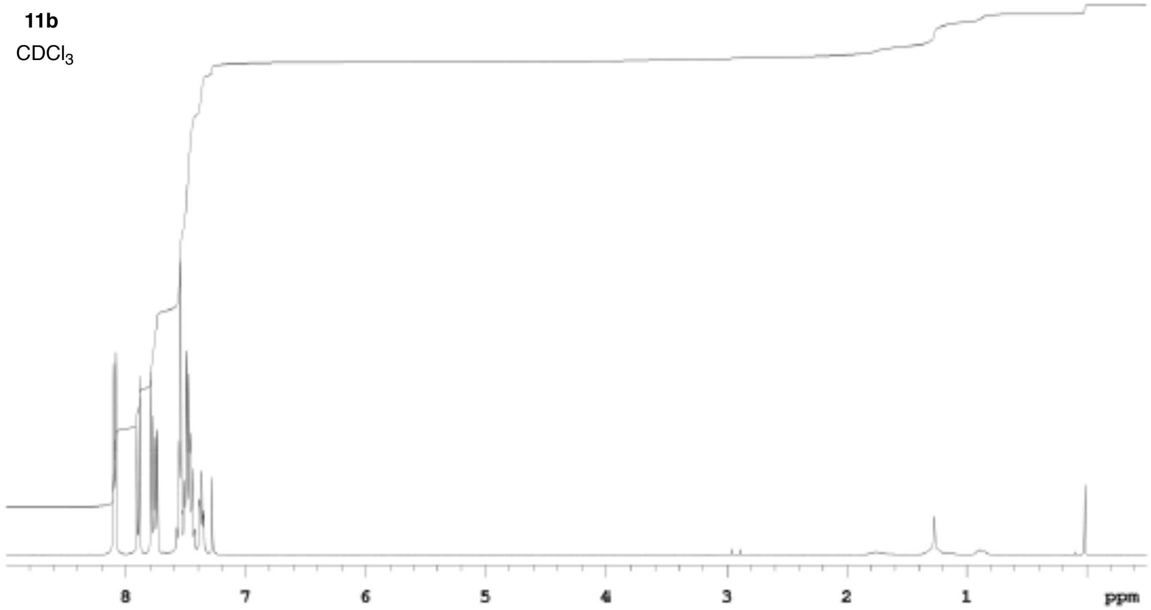

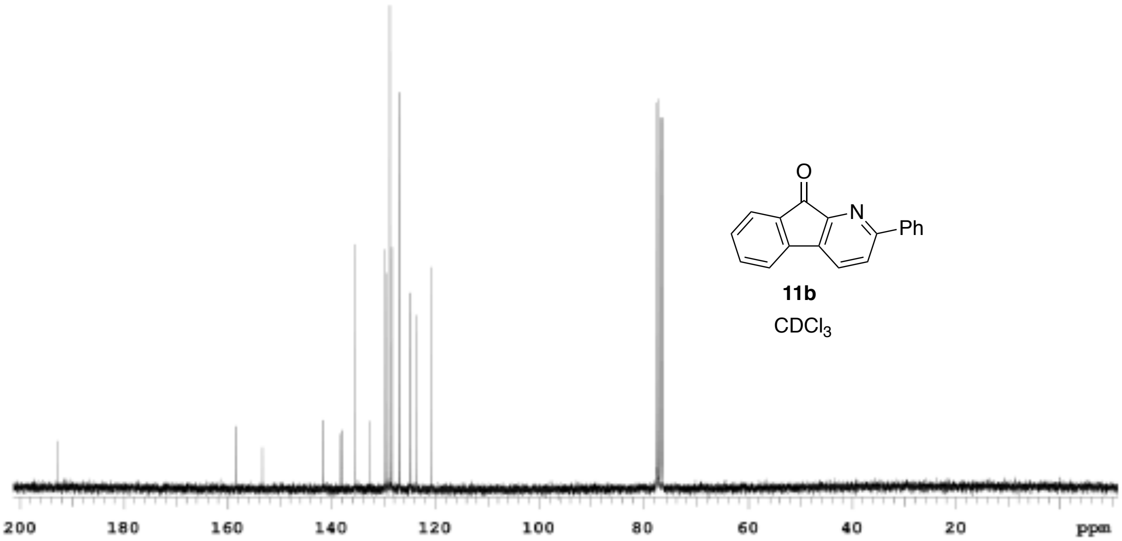

11b
$CDCl_3$

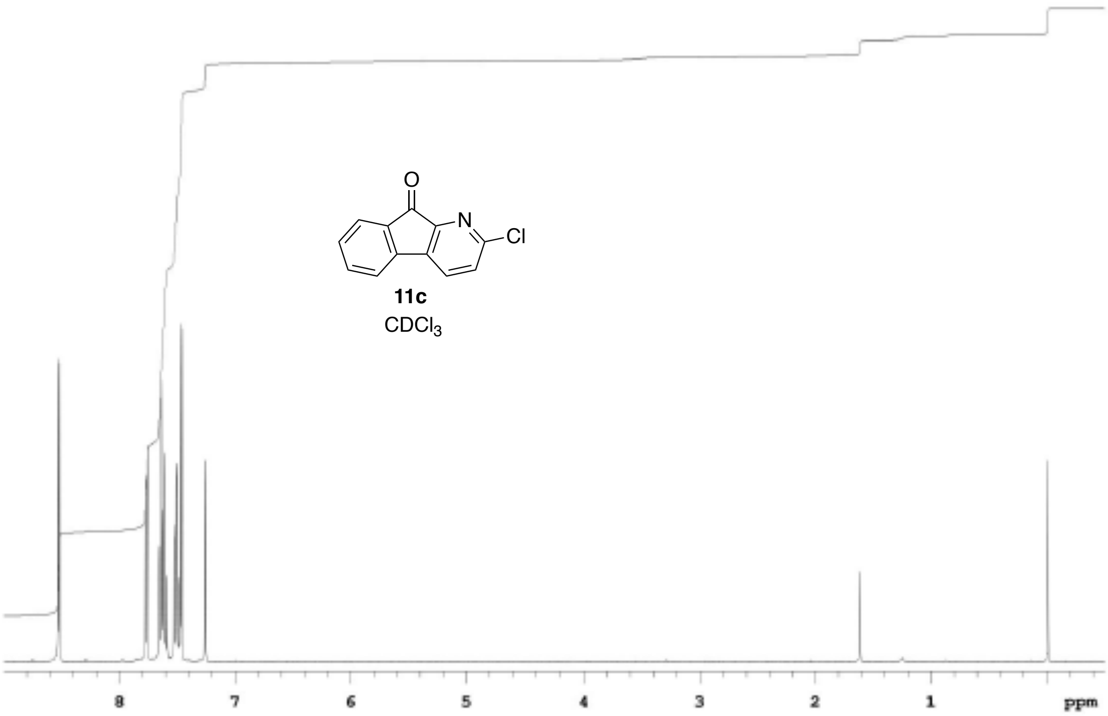
O
N
Cl
11c
$CDCl_3$
8
7
6
5
4
3
2
1
ppm

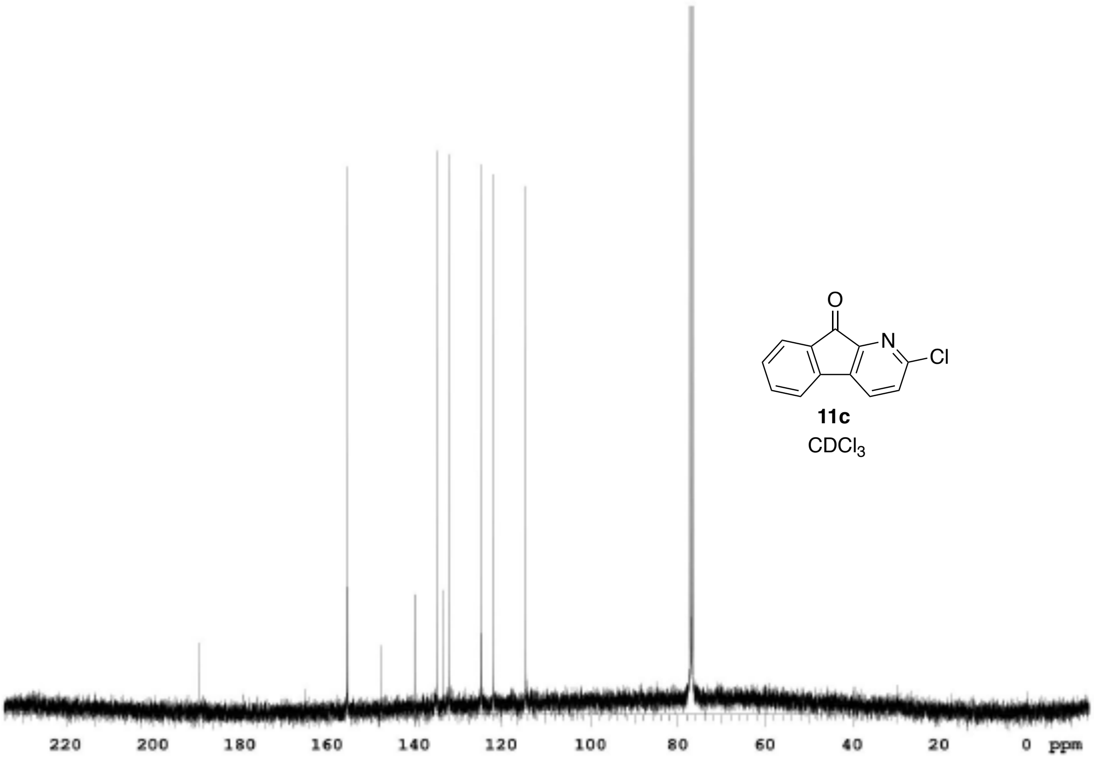
O
N
Cl
11c
$CDCl_3$
220
200
180
160
140
120
100
80
60
40
20
0
ppm
